# Automated detection of a nonperfusion area caused by retinal vein occlusion in optical coherence tomography angiography images using deep learning

**DOI:** 10.1371/journal.pone.0223965

**Published:** 2019-11-07

**Authors:** Daisuke Nagasato, Hitoshi Tabuchi, Hiroki Masumoto, Hiroki Enno, Naofumi Ishitobi, Masahiro Kameoka, Masanori Niki, Yoshinori Mitamura

**Affiliations:** 1 Department of Ophthalmology, Tsukazaki Hospital, Himeji, Japan; 2 Rist Incorporated, Tokyo, Japan; 3 Department of Ophthalmology, Institute of Biomedical Sciences, Tokushima University Graduate School, Tokushima, Japan; Newcastle University, UNITED KINGDOM

## Abstract

We aimed to assess the ability of deep learning (DL) and support vector machine (SVM) to detect a nonperfusion area (NPA) caused by retinal vein occlusion (RVO) with optical coherence tomography angiography (OCTA) images. The study included 322 OCTA images (normal: 148; NPA owing to RVO: 174 [128 branch RVO images and 46 central RVO images]). Training to construct the DL model using deep convolutional neural network (DNN) algorithms was provided using OCTA images. The SVM used a scikit-learn library with a radial basis function kernel. The area under the curve (AUC), sensitivity and specificity for detecting an NPA were examined. We compared the diagnostic ability (sensitivity, specificity and average required time) between the DNN, SVM and seven ophthalmologists. Heat maps were generated. With regard to the DNN, the mean AUC, sensitivity, specificity and average required time for distinguishing RVO OCTA images with an NPA from normal OCTA images were 0.986, 93.7%, 97.3% and 176.9 s, respectively. With regard to SVM, the mean AUC, sensitivity, and specificity were 0.880, 79.3%, and 81.1%, respectively. With regard to the seven ophthalmologists, the mean AUC, sensitivity, specificity and average required time were 0.962, 90.8%, 89.2%, and 700.6 s, respectively. The DNN focused on the foveal avascular zone and NPA in heat maps. The performance of the DNN was significantly better than that of SVM in all parameters (p < 0.01, all) and that of the ophthalmologists in AUC and specificity (*p* < 0.01, all). The combination of DL and OCTA images had high accuracy for the detection of an NPA, and it might be useful in clinical practice and retinal screening.

## Introduction

Retinal vein occlusion (RVO) is the second most common retinal vascular disease after diabetic retinopathy. Worldwide, the estimated number of RVO patients is 16.4 million [1], with a prevalence of 2.1% in the general population over 40 years of age [2], and risk factors include hypertension, diabetes and hyperlipidemia. RVO is a common cause of visual reduction from complications such as macular edema (ME), retinal bleeding and retinal ischemia [3,4]. RVO is divided into the following two types according to the occlusion site: branch retinal vein occlusion (BRVO) and central retinal vein occlusion (CRVO). Major vein occlusion of the retinal circulation can cause increased intraluminal pressure, hemorrhage and ME [5]. In recent years, intravitreal anti-VEGF agents have become the common clinical therapy for ME associated with BRVO and CRVO. In fact, numerous large-scale studies have reported that intravitreal injections of anti-VEGF agents significantly improve visual and anatomic outcomes for BRVO and CRVO patients with ME [6–12]. Although ME is most commonly associated with vision loss, thrombosis can result in engorged veins frequently accompanied by variable amounts of retinal nonperfusion.

**Fig 1:**
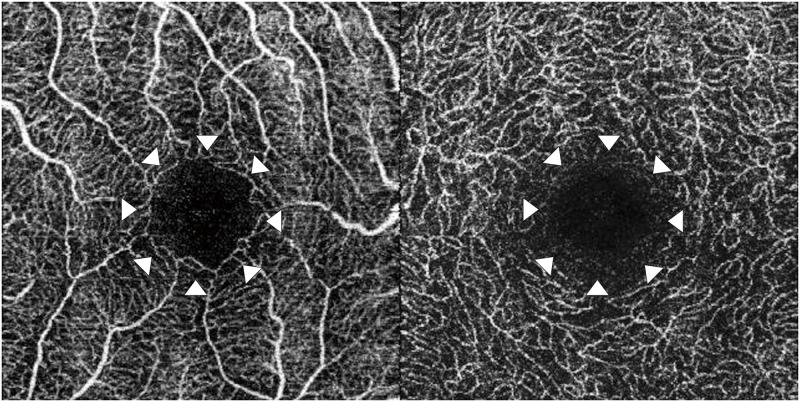
Representative images of the normal macula obtained using optical coherence tomography angiography (OCTA). The left image is a superficial capillary plexus OCTA image with a normal macula, and the right image is a deep capillary plexus OCTA image with a normal macula. The arrowheads indicate the foveal avascular zone.

**Fig 2:**
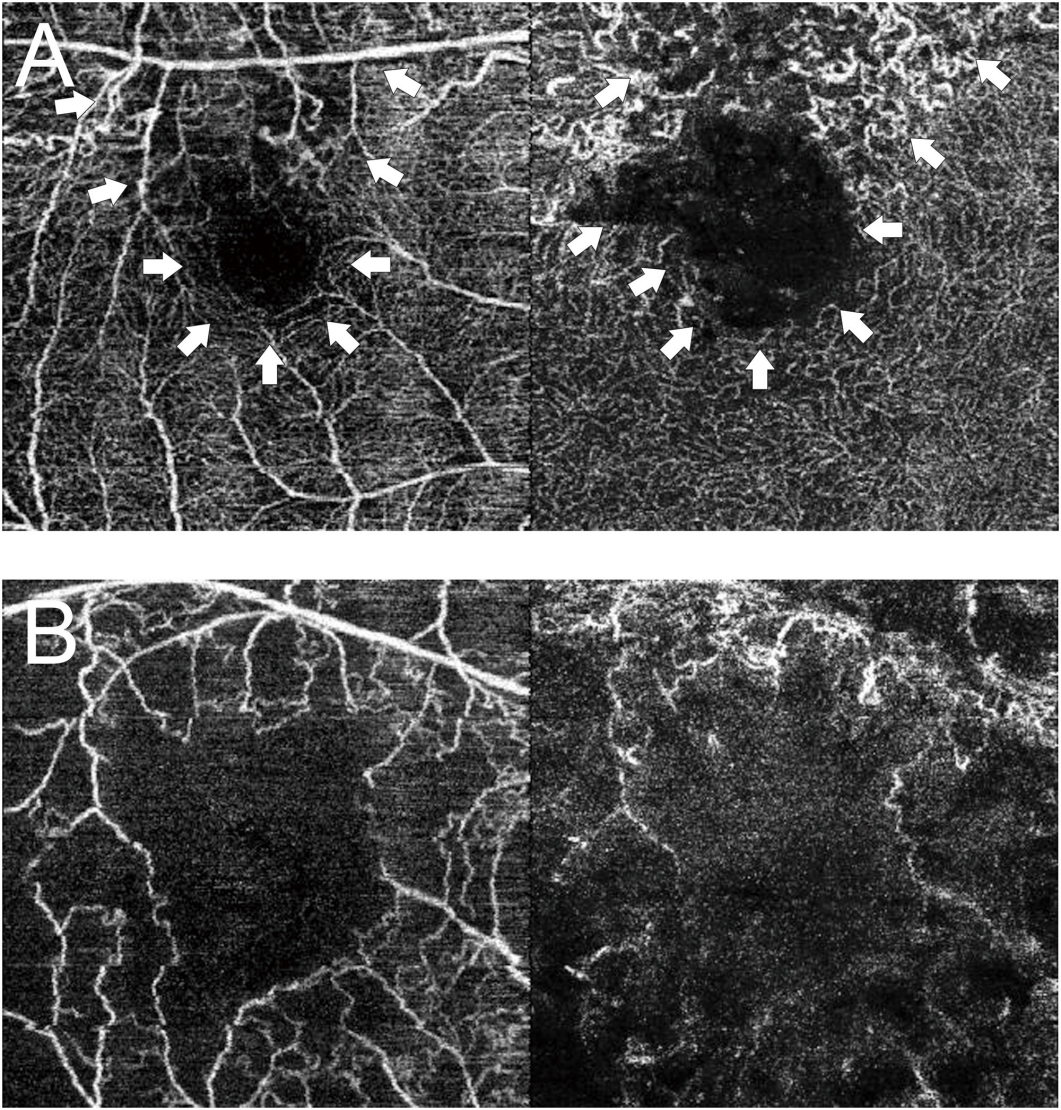
Representative retinal vein occlusion images of the macula obtained using optical coherence tomography angiography (OCTA). (A) The left image is the superficial capillary plexus (SCP) OCTA image with branch retinal vein occlusion (BRVO), and the right image is the deep capillary plexus (DCP) OCTA image with BRVO. The arrows indicate the foveal avascular zone and nonperfusion area with BRVO. (B) The left image is the SCP OCTA image with central retinal vein occlusion (CRVO), and the right image is the DCP OCTA image with CRVO. In the SCP and DCP OCTA images with CRVO, the foveal avascular zone and the nonperfusion area are observed throughout the cropped images.

Previously, angiography, including fluorescein angiography, was essential for diagnosing retinal vascular lesions. However, since angiography is an invasive examination, frequent examination is difficult. Additionally, visualizing the fine structure at the capillary level is difficult in these angiography images. Moreover, these images are two-dimensional images and cannot be assessed by stratification of the retina and choroid. In recent years, optical coherence tomography angiography (OCTA) has been devised, which can noninvasively detect a moving part of the fundus equivalent to red blood cells in the blood flow as a flow signal and visualize it as a blood vessel [13–16]. OCTA can analyze the retina in detail by dividing it into superficial capillary plexus (SCP) and deep capillary plexus (DCP) (Fig 1). Additionally, one report considered the foveal avascular zone (FAZ) and vessel density drawn from those images as a quantitative index [15]. Furthermore, the area of FAZ and visual acuity are reportedly inversely correlated in RVO and diabetic retinopathy (Fig 2) [17].

Recently, an image processing technology using deep learning (DL) and support vector machine (SVM), a machine-learning method, has been dramatically developed. According to several studies, image processing technology has very high classification performance in medical imaging [18–28]. In the ophthalmology field, recent investigations have demonstrated the application of image processing technology involving machine-learning algorithms in medical imaging for various retinal diseases, including BRVO and CRVO, using fundus color photographs and ultra-widefield fundus ophthalmoscopy images [21,23–25,29–32]. In a recent investigation, DL segmented the nonperfusion area (NPA) in OCTA images of diabetic retinopathy [33].

However, to the best of our knowledge, no studies have focused on the automated diagnostic accuracy of image processing technology involving DL and SVM for the NPA using OCTA images of RVO.

Thus, the present study aimed to assess the ability of image processing technology involving DL and SVM to detect an NPA owing to RVO using OCTA images. This study was performed at Tsukazaki Hospital and Tokushima University Hospital.

## Materials and methods

### Data set

The OCTA images of normal eyes and eyes with NPA caused by RVO were extracted from the clinical databases of the Ophthalmology departments of Tsukazaki Hospital and Tokushima University Hospital. A retinal specialist reviewed and confirmed the presence of NPA by assessing 3 × 3 mm OCTA images for the SCP and DCP. The OCTA images were then registered on a database for analysis. There were 322 OCTA images included in the current study. With regard to BRVO, eyes without NPA in OCTA images were not included. NPA with CRVO was present in all eyes. To assess OCTA image processing accuracy with DL for an NPA, we focused on the OCTA images of normal and NPA with acute RVO cases. We did not include NPA cases with chronic RVO in which abnormal conditions, such as collateral vessels, may exist in addition to NPA. Moreover, NPA cases with diabetic retinopathy were not included in which other abnormal conditions, such as microaneurysms, must be considered. These additional abnormalities may be confounders that make it difficult for DL to determine the NPA.

In the current study, we used *K*-fold cross-validation (*K* = 8), which was previously reported [34,35]. In brief, OCTA imaging data were divided into *K* groups. Then, (*K*−1) groups were used for training, and one group were used for validation. This process was repeated *K* times until each of the *K* groups reached the validation data set.

The OCTA images in the training data set were augmented with image transformation processes such as brightness adjustment, gamma correction, histogram equalization, and noise addition and inversion. The amount of training images approached 18 times the amount of original training data. A deep convolutional neural network (DNN) model, as described below, was created and trained with the augmented training data. These processes are described in supplemental files (in data_augment.py).

Because of the retrospective and observational nature of the study, the need for written informed consent was waived by the ethics committees. The data acquired in the course of the data analysis were anonymized before we accessed them. This study adhered to the tenets of the Declaration of Helsinki, and it was approved by the local ethics committees of Tsukazaki Hospital and Tokushima University Hospital.

### Deep-learning model and training

**Fig 3:**
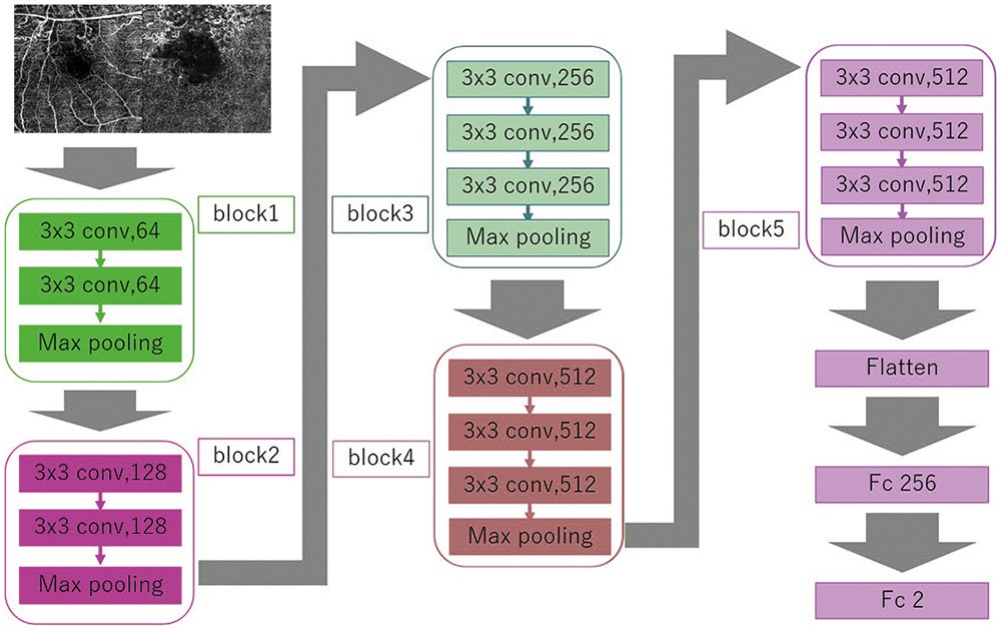
Overall architecture of the Visual Geometry Group (VGG)-16 model. A data set of resized optical coherence tomography angiography images (256 × 192 pixels) is the input. VGG-16 includes five blocks and three fully connected layers. Each block includes some convolutional layers followed by a max-pooling layer. The output of block 5 is flattened, resulting in two fully connected layers. The first layer removes spatial information from the extracted feature vectors, and the second layer is a classification layer that uses the feature vectors of the target images acquired in previous layers and the softmax function for binary classification.

We implemented a DL model that uses a Visual Geometry Group (VGG)-16 DNN (Fig 3). This DNN automatically learns local features of images and generates a classification model [36–38]. The input in this study was concatenated OCTA images of SCP and DCP images. The size of the concatenated original OCTA images was 640 × 320 pixels. We converted the size of the original input images to 256 × 192 pixels because of the reduction in the analysis time. The RGB image input had a range of 0 to 255, and the input was first normalized to a range of 0 to 1 by dividing the values by 255. The shape of the input tensors used in this study is 256 × 192 × 3.

The VGG-16 DNN included five blocks and three fully connected layers. Each block included some convolutional layers followed by a max-pooling layer, which decreased positional sensitivity, improving generic recognition [39]. These convolutional layers capture only the features of the image without shrinking because the strides of convolution layers were 1 and the padding of the layers were the “same”. We could avoid the vanishing gradient problem because the activation function of the layers was ReLU [40]. The strides of max-pooling layers were 2, so these layers compressed the information of the image. The output of block 5 was flattened, and subsequently, two layers were fully connected. The first layer removed spatial information from the extracted feature vectors, and the second layer was a classification layer that used the feature vectors of the target images acquired in previous layers and the softmax function for binary classification. To improve generalization performance, we carried out a dropout process to mask the first fully connected layer with 25% probability. The output of the neural network was the vector of order 2 representing probability for each class value (non-RVO, RVO).

Fine tuning was applied to increase the learning speed for high performance achievement even with limited data [41,42]. We used parameters from ImageNet as initial parameters of blocks 1 to 5.

The layers were updated using the optimization momentum stochastic gradient descent algorithm (learning rate = 0.0005, momentum coefficient = 0.9) [43,44]. Mini Bach size was 32. Among the 20 DL models obtained in 20 learning cycles, the model with the highest correct answer rate for the available test data was selected as the final DL model in each split. To build and evaluate the model, we ran Keras (https://keras.io/ja/) on TensorFlow (https://www.tensorflow.org/), which was written in Python.

### Support vector machine model

We used the soft-margin SVM implemented in the scikit-learn library using the radial basis function kernel [45]. We reduced all images to 10 dimensions. Optimal values for cost parameter “C” of the SVM algorithm and parameter “γ” of the radial basis function were determined by grid search using quadrant cross-validation, and the combination with the highest accuracy was selected in each split. The parameter values tested for C were 1, 10, 100, and 1000, and those for γ were 0.0001, 0.001, 0.01, 0.1, and 1. The optimized parameter values of C and γ in each split are described in S1 File.

### Outcome

The area under the curve (AUC) of the receiver operating characteristic curve, sensitivity, and specificity were determined from the concatenated OCTA images using the DNN and SVM model described above.

### Creation of the test application for ophthalmologist interpretation

We compared the diagnostic accuracy between the DNN and ophthalmologists. All 322 concatenated OCTA images were included. The sensitivity, specificity, and required time were determined for the DNN and seven ophthalmologists. Details were shown in S2 File.

### NPA assessment and required time

The seven ophthalmologists assessed the presence or absence of an NPA by reviewing the 322 concatenated OCTA images as indicated on the computer screen, without other images. Using a Microsoft Excel-based response form, each of the seven ophthalmologists entered the integer 0 or 1 directly into a computer. Details were shown in S3 File.

### Statistical analysis

With regard to background demographic data, Student’s *t*-test was used to compare age, and Fisher’s exact test was used to compare the ratios of gender and left/right affected eyes between patients and normal subjects. These statistical analyses were performed using Python Scipy (https://www.scipy.org/), Python Statsmodels (http://www.statsmodels.org/stable/index.html) and R pROC (https://cran.r-project.org/web/packages/pROC/pROC.pdf). A *p* value of <0.05 was considered statistically significant.

The 95% confidence interval (CI) of the AUC was obtained as follows. The OCTA images judged to exceed a threshold were considered positive for RVO, and a receiver operating characteristic (ROC) curve was created. For the AUC, the 95% CI was obtained by assuming a normal distribution and calculated in these equations [46].

95%CIofAUC=AUC+Z(0.05/2)*SE(AUC)

$$Z(x)=\frac{1}{\sqrt{2\pi }}e^{-\frac{x^{2}}{2}}$$

$$SE(AUC)=\sqrt{\frac{AUC*(1-AUC)+(n_{p}-1)*(Q_{1}-{AUC}^{2})+(n_{n}-1)*(Q_{2}-{AUC}^{2})}{n_{p}*n_{n}}}$$

$$Q_{1}=\frac{AUC}{2-AUC}$$

$$Q_{2}=\frac{{2AUC}^{2}}{1+AUC}$$

*n*_*p*_ … the number of RVO images, 174

*n*_*n*_ … the number of normal images, 148

In the RVO classification, in sensitivity and specificity image output by the neural network higher than 0.5 was classified as RVO, and image output lower than 0.5 was classified as normal. Additionally, regarding the sensitivity and specificity of seven ophthalmologists, if four or more ophthalmologists considered OCTA images positive, these images were considered positive. The 95% CIs of sensitivity and specificity were calculated assuming a binomial distribution. Fleiss’ kappa coefficients were used to assess the agreement rate among seven ophthalmologists for NPA detection [47,48]. Fisher’s exact test was used to compare the sensitivity and specificity between the DNN, SVM and ophthalmologists.

### Heat map

Overlaying heatmap images of the DNN focus site were created using a gradient-weighted class activation mapping (Grad-CAM) method on the corresponding RVO and non-RVO OCTA images [49]. In the current study, we used the grad-CAM method to maximize the outputs of the second convolution layer in block 2. The function in the backpropagation steps for modification of the loss function was a rectified linear unit, which propagated only positive gradients. This process was performed using Python Keras-vis (https://raghakot.github.io/keras-vis/).

## Results

The study included 322 OCTA images. Of these images, 174 were of eyes with NPA owing to RVO [170 patients (mean age: 71.4 ± 10.9 years); 90 eyes from men and 84 from women; 79 left and 95 right eyes; and 128 eyes with BRVO and 46 with CRVO], and 148 images were of normal eyes [147 subjects (mean age: 70.4 ± 10.8 years); 75 eyes from men and 73 from women; and 81 left and 67 right eyes]. No significant differences were detected between these two groups with respect to age, gender ratio, and left-right eye image ratio (*p* = 0.401, *p* = 0.911, and *p* = 0.117, respectively) (Table 1).

**Table 1:** Comparison of demographic variables between the nonperfusion area owing to retinal vein occlusion and normal groups.

	NPA owing to RVO group	Normal group	P value
Number of images	174	148	
Patients	170	147	
Women (%)	84 (48.3)	73 (49.3)	0.911*
Mean age (SD)	71.4 (10.9)	70.4 (10.8)	0.401**
Left fundus (%)	79 (45.4)	81 (54.7)	0.117*

NPA, nonperfusion area; RVO, retinal vein occlusion; SD, standard deviation.

* Fisher’s exact test.

** Student’s *t*-test.

**Fig 4:**
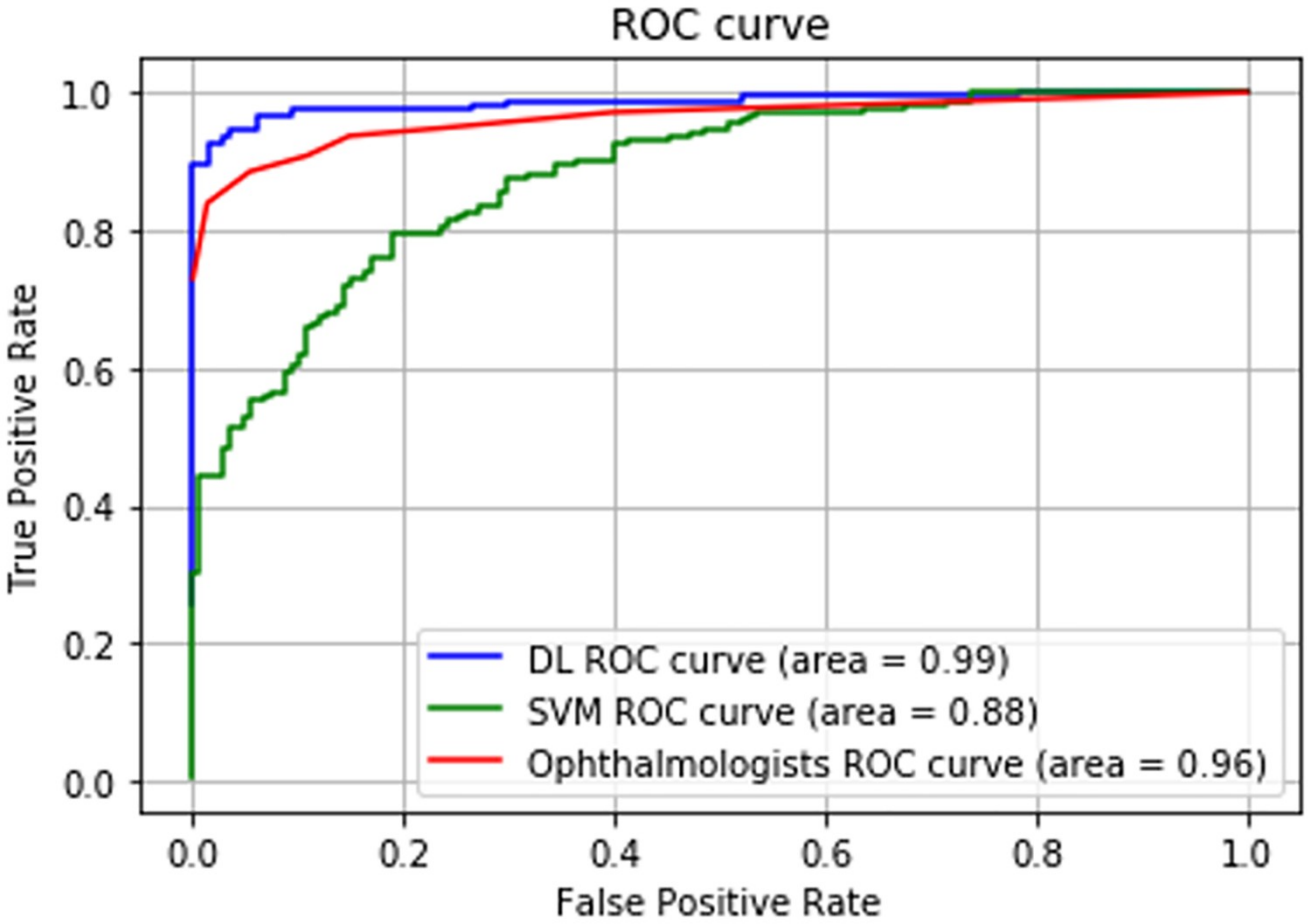
The Receiver operating characteristic curves in the deep-learning model, support vector machine model and ophthalmologists. The area under the curve is 0.986 in the deep-learning model, 0.880 in the support vector machine model and 0.962 in the seven ophthalmologists.

With regard to the detection of an NPA owing to RVO, the DNN had a sensitivity of 93.7% (95% CI, 89.0–96.8%), specificity of 97.3% (95% CI, 93.2–99.3%), AUC of 0.986 (95% CI, 0.974–0.999) and average required time of 176.9 s (95% CI, 172.4–180.2 s). The SVM had a sensitivity of 79.3% (95% CI, 72.5–85.1%), specificity of 81.1% (95% CI, 73.8–87.0%) and AUC of 0.880 (95% CI, 0.843–0.918) (Fig 4). The ophthalmologists had a sensitivity of 90.8% (95% CI, 85.5–94.7%), specificity of 89.2% (95% CI, 83.0–93.7%), AUC of 0.962 (95% CI, 0.942–0.983) and average required time of 700.6 s (95% CI, 585.2–816.0 s) (Table 2). The mean kappa coefficient among seven ophthalmologists for the detection of an NPA was 0.746 (95% CI, 0.725–0.766).

**Table 2:** Comparison of the abilities of the deep convolutional neural network, support vector machine and ophthalmologists (n = 7) to detect a nonperfusion area.

	DL (95% CI)	SVM (95% CI)	Ophthalmologists (95% CI)	P value (DNN vs SVM)	P value (DNN vs Ophthalmologists)
Sensitivity (%)	93.7 (89.0–96.8)	79.3 (72.5–85.1)	90.8 (85.5–94.7)	<0.01*	0.42*
Specificity (%)	97.3 (93.2–99.3)	81.1 (73.8–87.0)	89.2 (83.0–93.7)	<0.01*	<0.01*
AUC	0.986 (0.974–0.999)	0.880 (0.843–0.918)	0.962 (0.942–0.983)	<0.01*	<0.01*
Average required time (sec)	176.9 (172.4–180.2)		700.6 (585.2–816.0)		<0.01*

AUC, area under the curve; CI, confidence interval; DL, deep learning; SVM, support vector machine.

* Fisher’s exact test.

**Fig 5:**
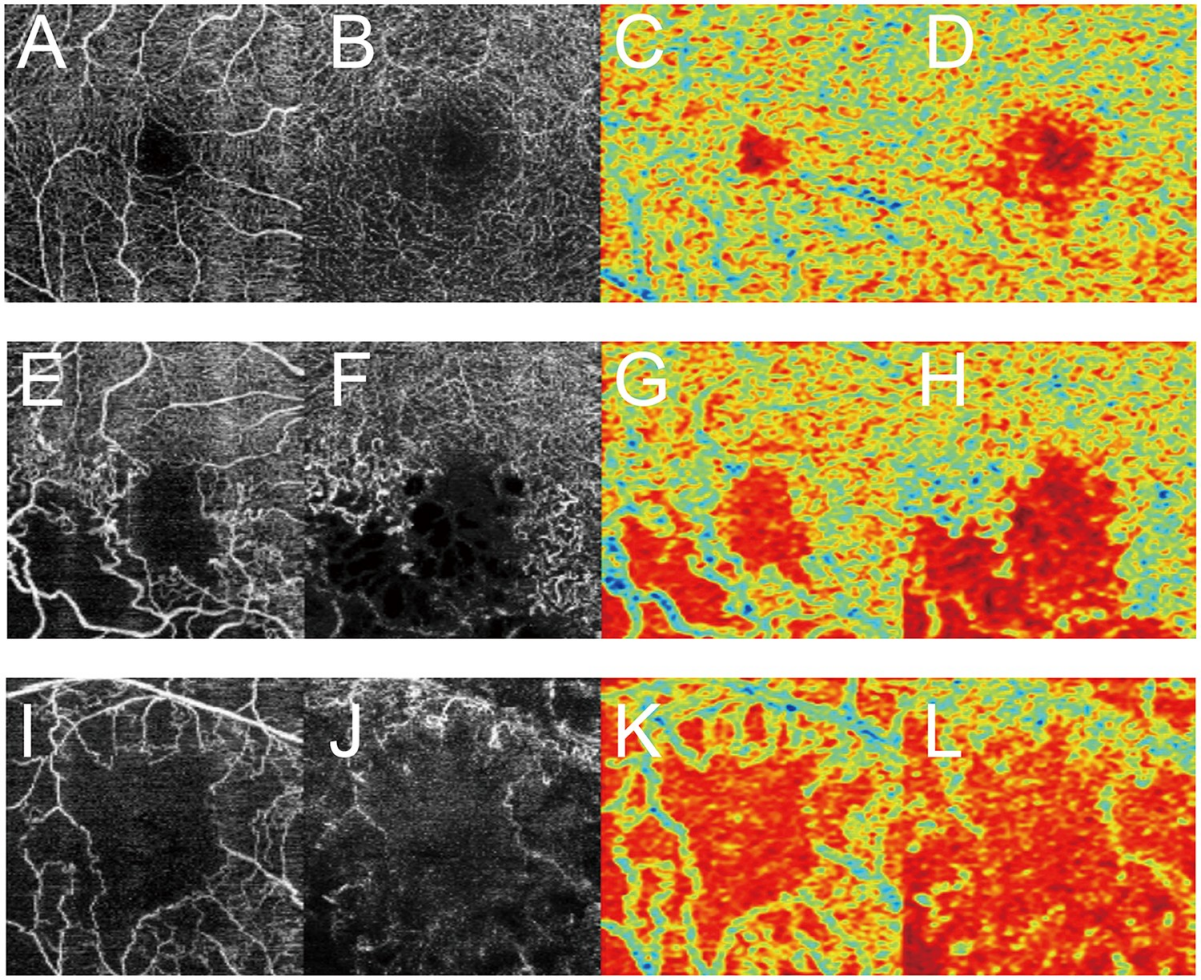
Representative images obtained using optical coherence tomography angiography (OCTA) and their heat maps. (A) Normal superficial capillary plexus (SCP) OCTA image, (B) normal deep capillary plexus (DCP) OCTA image, (C) heat map of the normal SCP OCTA image, (D) heat map of the normal DCP OCTA image, (E) SCP OCTA image with a nonperfusion area (NPA) owing to branch retinal vein occlusion (BRVO), (F) DCP OCTA image with an NPA owing to BRVO, (G) heat map of the SCP OCTA image with BRVO, (H) heat map of the DCP OCTA image with BRVO, (I) SCP OCTA image with an NPA owing to central retinal vein occlusion (CRVO), (J) DCP OCTA image with an NPA owing to CRVO, (K) heat map of the SCP OCTA image with CRVO and (L) heat map of the DCP OCTA image with CRVO. Red is used to indicate the strength of deep convolutional neural network focus. The color intensity is high at the area of the foveal avascular zone and NPA in SCP and DCP OCTA images; accumulation is noted at the focal points. The deep convolutional neural network focused on the foveal avascular zone and NPA.

A composite image, comprising the fundus image superimposed with its corresponding heat map, was created by the DNN, and these images showed that DNNs could accurately identify crucial areas in the fundus images; a representative composite image is presented in Fig 5. Blue indicates the strength of DNN-based identification, and an increase in color intensity was observed in areas with the FAZ area and NPA at the fovea in SCP and DCP OCTA images at the focal points. These results imply that the DNN might differentiate RVO eyes from normal eyes by identifying and highlighting the NPA. Red indicates the strength of DNN focus. The color intensity was high in the FAZ area and NPA in the SCP and DCP OCTA images. Accumulation occurred in focal points.

## Discussion

Generally, FA is considered the gold standard for diagnosing and delineating the extent of retinal ischemia. However, the recent emergence of three-dimensional, noninvasive imaging using OCTA has provided an opportunity to quantify vessel density in a defined retinal area and to gage its loss over time, either physiologically with aging or through an underlying vascular pathology [50–52]. Recent studies have identified measurable parameters, such as vessel density, capillary length, intercapillary distance and FAZ area, to quantify the degree of retinal ischemia and to longitudinally assess its progression [23,53–55].

With regard to the representation of the NPA and the FAZ area associated with RVO, OCTA images are clearer than FA images, and the boundary in OCTA images is clear [15,16]. In the present study, the performance of the DNN was significantly better than that of SVM in all parameters (p < 0.01) and that of ophthalmologists in the specificity, AUC and average required time (p < 0.01). The combination of DL and OCTA images had high accuracy for the detection of an NPA, and it might be useful in clinical practice and retinal screening. Recent investigations have demonstrated a high AUC for detecting diabetic retinopathy on retinal fundus photography [21,29] and rhegmatogenous retinal detachment on ultra-widefield fundus ophthalmoscopy [30]. Moreover, in the radiological field, it has been proposed that perfusion image quality is better and perfusion measurement is more accurate with convolutional neural network techniques, such as a DL algorithm, than with the conventional averaging method for the generation of arterial spin labeling images from pairwise subtraction images [56]. In the present study, the AUC and required time for distinguishing between normal eyes and NPA owing to RVO eyes were better with the DNN than with ophthalmologist assessment. Guo et al. [33] reported that the NPA in OCTA images of diabetic retinopathy was segmented by DL. However, these authors detected NPA in OCTA images using a manually segmented nonperfusion binary map. In our study, we did not use manual images to detect NPA in OCTA images. The NPA in OCTA images was relatively clear as we used images with a narrow angle of view, and DL easily distinguished NPA OCTA images from normal OCTA images.

Retinal ischemia is a key prognostic factor in the management of various retinal diseases, including RVO. Several studies have demonstrated that decreases in both the SCP and DCP vessel density, fractal dimension and skeletal vessel density on OCTA are associated with RVO severity [16,17,57]. In fact, according to the heat maps, the DNN focused on the FAZ area in normal SCP and DCP OCTA images and the FAZ area and NPA in RVO SCP and DCP OCTA images. Our results indicate that the DNN has a classification ability that is equivalent to or greater than the ability of ophthalmologists. Therefore, the identification of an NPA using DL and OCTA is considered highly useful and clinically significant. The ability of DL to distinguish between RVO and normal eyes with high accuracy using automatically segmented OCTA images suggests the possibility of automatic diagnosis of eye disease by artificial intelligence in the future.

The present study had some limitations. First, we compared only OCTA images between normal eyes and RVO eyes and did not include OCTA images of other retinal diseases. Further studies involving other retinal diseases are required to confirm our findings. Additionally, for extensive evaluation of the performance and versatility of DL for the detection of an NPA, it will be necessary to use larger samples and include OCTA images of other retinal diseases. Second, the scan area of 3 × 3 mm was not large enough to detect the entire NPA associated with RVO. Wider ranges of examination areas may provide more conclusive evidence.

## Conclusions

In conclusion, the combination of DL and OCTA images had high accuracy for the detection of an NPA. DL was useful for detecting NPA in OCTA images. These findings suggest that further investigations are required to develop artificial intelligence that detects retinal ischemic disorders.

## Supporting information

S1 File The optimized parameter values of cost and gamma in each split. (XLSX)

S2 File The diagnostic accuracy between the deep convolutional neural network and ophthalmologists. (CSV)

S3 File The seven ophthalmologists assessed the presence or absence of the non perfusion area. (CSV)

S1 Data Set (ZIP)
